# The shell shape optimization and fluid–structure interaction simulation of hose pump in water-fertilizer integrated fertilizer application

**DOI:** 10.1038/s41598-022-07273-6

**Published:** 2022-02-28

**Authors:** Xiao Ma, Lixin Zhang, Wendong Wang, Yongchun Yan, Chanchan Du

**Affiliations:** College of Mechanical and Electrical Engineering, Shihezi University, Beisi Road, Shihezi, 832003 Xinjiang China

**Keywords:** Mechanical engineering, Applied mathematics

## Abstract

As a new type of under-film drip irrigation, water-fertilizer integrated fertilizer application device in Northwest China, the hose pump has achieved excellent results in practical applications, but its pulsation has exhibited some adverse effects on the fertilization process. By analyzing the cause of pulsation and flow characteristics, we proposed a shell optimization method to reduce pulsation. We used a release time deformation curve as the shape curve of the outlet shell of the hose pump. Based on the fluid–structure interaction analysis, we developed a numerical model of an optimized three roller hose pump and a conventional three roller hose pump for dynamic simulations. The simulation results showed the optimized hose pump flow pressure variation range was reduced by 26.92%, the average fluid flow velocity increased by about 10%, and mass flow rate improved by 8.84% over the conventional hose pump. We tested the optimized hose pump prototype and the conventional hose pump on the test bench. The test results showed that the pulsating pressure variation range of the optimized pump decreased by about 20%, and flow output increased by about 8.63%. These results suggest that shell shape optimization assist in the decrease of flow pulsation and contribute to further hose pump popularization.

## Introduction

In northwestern China, based on climatic conditions and planting characteristics, under-film drip irrigation with water and fertilizer integration is widely used in agriculture^1^. Water-fertilizer integrated fertilizer application devices include the venturi fertilizer applicator, differential pressure fertilization tank, and pump fertilization device^2^. Nowadays, pump fertilizer application devices are increasingly used due to their effective power characteristics and ease of precise control^3,4^. Hose pumps belong to the peristaltic pumps category, which is a kind of rotor type volumetric pump^5,6^. The hose pump exhibits effective self-priming performance and performs well in actual use, but the pulsation phenomenon occurs. The pulsation phenomenon has some influence on the pump body and also has adverse effects on the water-fertilizer integrated fertilizer application process^7–9^.

Some studies have been conducted on the flow characteristics, pulsation characteristics, and numerical simulations of hose pumps. Natarajan and Mokhtarzadeh-Dehghan^10^ present a numerical study of the flow field in a novel “soft” acting peristaltic pump, and the results show the flow has generally a two-way pulsatile nature, moving forwards and backwards. Formato^11^ applied fluid–structure interaction (FSI) modeling for predicting the fluid flow in a specific peristaltic pump; hyper-elastic material dynamics and turbulence flow dynamics were coupled to describe all the physics of the pump, and the applicability of FSI modeling for geometric optimization of pump housing was also discussed to prevent roller and hose parts pressure peaks. Zhou^12^ presented a generalized parametric model for the fluid field in a typical roller pump system and deduced analytical formulations of the moving boundary. Based on the model and formulations, the dynamic geometry and mesh of the fluid field can be updated automatically according to the time-dependent roller positions. The described method successfully simulates the pulsing flow generated by the pump. Elabbasi^13^ used the 3D FSI of COMSOL multi-physics to study the performance of a 180-degree rotating peristaltic pump with two metal rollers, accurately designed the flow parameters of a peristaltic pump, and carried out theoretical calculations and experimental verifications. Wang^14^ established a three-dimensional, two-way fluid structure coupling model of the hose pump to study its pressure fluctuation characteristics. The results show the roller pass frequency is the main frequency of the pressure fluctuation at the outlet of the hose pump.

To reduce the influence of the pulsation phenomenon, we first analyze the pulsation reason and flow characteristics of hose pumps then propose a hose pump shell shape optimization method. Based on fluid–structure interaction (FSI) analysis, we developed the numerical model of the optimized three roller hose pump and the conventional three roller hose pump to dynamic simulations. To verify the practical application effect, we tested the optimized hose pump prototype and the conventional hose pump on the test bench.

## Analysis of pulsation causes and flow characteristics

### Analysis of pulsation causes

**Figure 1:**
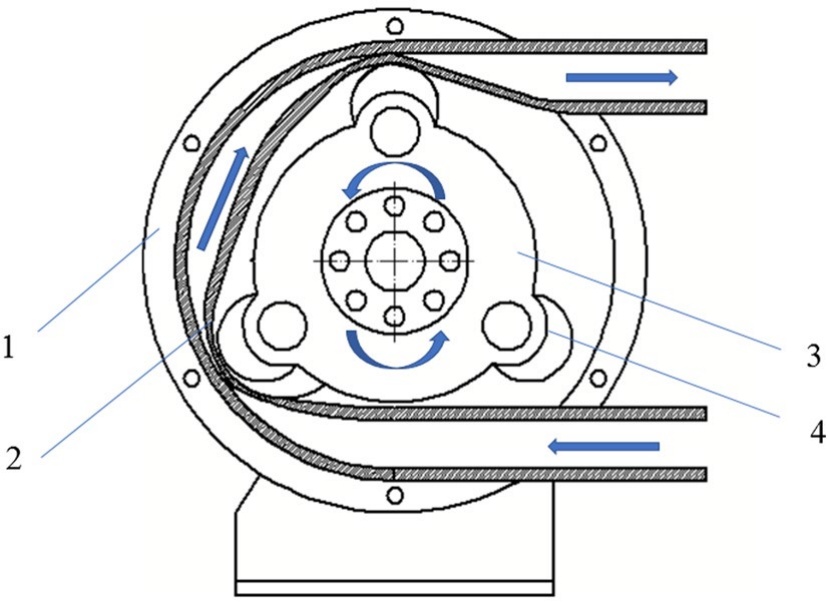
Working principle diagram of the hose pump. 1—hose pump shell, 2—hose, 3—the rotating disc, 4—roller.

Take the conventional three roller hose pump as an example, as shown in Fig. 1. When the hose pump works, the rotating disc drives the roller to rotate, and the roller squeezes the hose fixed in the hose pump housing. The hose generates a vacuum under the action of its own elastic force, sucks and transports the liquid fertilizer liquid, and then the fertilizer liquid in the pipe moves forward with the roller. At the outlet of the hose pump, the roller releases the hose, and the fertilizer liquid is discharged from the outlet of the hose pump. Cycle back and forth to complete the transportation of the liquid fertilizer.

At the outlet position of the hose pump, the liquid fertilizer increases and decreases intermittently. The liquid in the pump tube does not flow at equal speed^15^; it changes periodically because, when the drum is about to release the hose, the pressure acting on the pump hose disappears rapidly, the liquid fertilizer flow space in the pump tube increases rapidly, the negative pressure is generated inside the hose, the liquid fertilizer at the outlet is sucked back, and then the recovery deformation of the pump tube makes part of the liquid flow back to the recovery deformation zone of the pump tube.

### Analysis of flow characteristics

**Figure 2:**
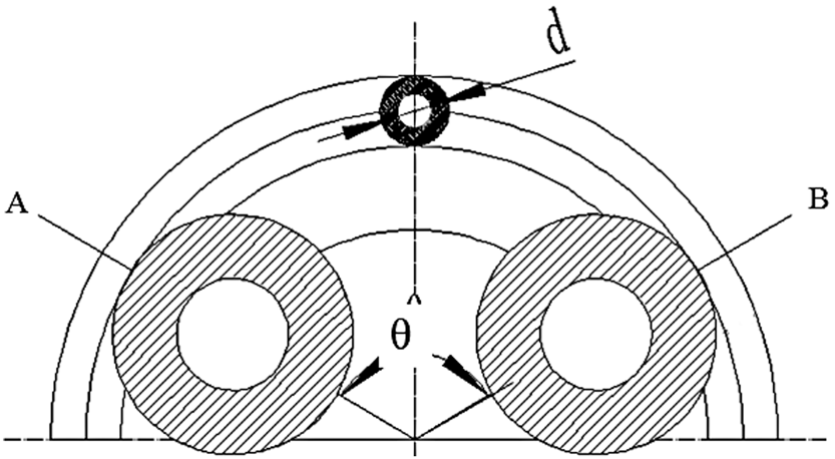
Schematic diagram of the hose pump flow principle.

The pulsation phenomenon complicates the calculation of the hose pump flow rate. Liquid fertilizer viscosity, hose pump joints, pipe pressure, hose material properties, and hose pump speed have an effect on hose pump flow^16^. Figure 2 shows a simplified model of a hose pump. In this model, the external factors of the system, such as liquid fertilizer viscosity, are not considered. The roller completely squeezes the hose from point A until it reaches point B, where the hose is released and the liquid in the hose is discharged. Without considering the volume occupied by the rollers rolling the pump tube, the volume of liquid delivered by the rollers from position A to position B is equal to the volume stored in the circular AB section of the pump tube.

Then

1 $$ \mathop \Delta \nolimits_{q} = \frac{\theta D}{2} \cdot \frac{{\pi d^{2} }}{4} = \frac{{\pi \theta Dd^{2} }}{8} $$

where $\mathop \Delta \nolimits_{q}$ represents the volume of liquid in the AB segment hose, in cubic centimeters; $\theta$ represents the angle at which the rotor of a hose pump rotates, in rad; *D* represents the diameter of the circumferential pitch circle of the pump shell, in millimeters; and *d* represents the inner diameter of the hose, in centimeters.

Therefore, the volume of the liquid transported by the hose pump rotating a circle is as follows:

2 $$ Q = \frac{2\pi }{\theta } \cdot \Delta q $$

Ideally, assuming the hose is not deformed, then

3 $$ Q = n \cdot Q = 2.467nDd^{2} $$

where *n* is the rotor speed, in rad/s.

In the actual working process, the hose deformation will inevitably occur when it is squeezed by the roller. The actual flow rate of the pump will be as follows:

4 $$ Q_{{\text{v}}} = n \cdot Q = 2.467nDd^{2} C_{d} $$

where $Q_{v}$ represents the actual flow in mL/s and $C_{d}$ represents the deformation coefficient of the hose, which is related to the hose material and generally in the 0.6–0.7 range.

Therefore, for the hose pump, without considering the volume occupied by the rollers compacting the pump pipe, the flow of the hose pump is independent of the number of rollers and other parameters, but it is related to the inner diameter of the pump pipe, the circumference diameter of the pump shell, and the rotation speed of the hose pump.

## The shell shape optimization of the hose pump

### Mathematical model analysis

We assume that the slower and more stable the hose pump roller releases the hose at the outlet, the smaller the pulsation of the medium output. The process of releasing the hose at the outlet by the pressure roller is studied in detail below^17–19^.

**Figure 3:**
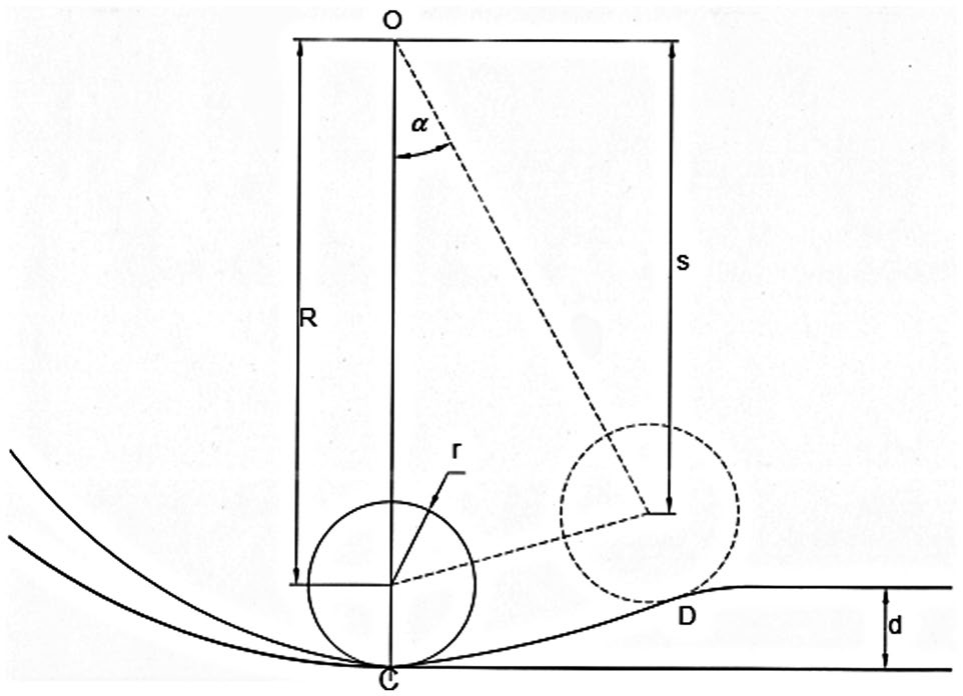
Mathematical model of the roller releasing hose.

As shown in Fig. 3, when the roller squeezes the hose to the maximum, the inside diameter of the hose is closed. During the release of the hose by the roller at the outlet of the pump, the longitudinal distance of the hose from point C to point D is *L*, and the release rotation angle is *α*, the release time is *t*, and the internal diameter of the hose is *d*, then when *α* from *0* turns to *α*_*max*_ (i.e., the release time changes from *0* to *t*), *l* will change from *0* to *d*.

We assume that the rated speed of the hose pump is *n*, the radius of the roller is *r*, the distance from the center of the pump body to the center of the roller is *s*,

Then

5 $$ s = OC - r - {\text{d}} = R - {\text{d}} $$

According to the trigonometric theorem,

6 $$ {{\cos}}\alpha_{{{\text{max}}}} = \frac{s}{R} = \frac{{R - {\text{d}}}}{R} $$

maximum release angle

7 $$ \alpha_{{{\text{max}}}} = {\text{arccos}}\left( {\frac{{R - {\text{d}}}}{R}} \right) $$

release time

8 $$ {\text{t}}_{{{\text{max}}}} = \frac{\alpha }{2\pi n} = \frac{{{\text{arccos}}\left( {\frac{{R - {\text{d}}}}{R}} \right)}}{2\pi n} $$

From the derivation of the mathematical equation, we can obtain the angle of rotation and the longitudinal distance of the process of releasing the hose from the roller. We found that the function L = f(*t*) is related to the distance from the center of the pump shell to the center of the roller, the inner diameter of the hose, and the velocity when determining the pump hose material. The function L = f(*t*) is independent of the radius of the roller.

The shell is an important contact part of extrusion movement. It is of great significance to improving the dynamic performance through shell optimization^20,21^. We assume that the curve of *L* with the release time *t* at rated speed is used as the shape curve of the hose pump outlet shell to compensate for the curve change of the hose release process in the pump, thus reducing the pulsation effect.

### The curve change of the hose release process in the pump

**Figure 4:**
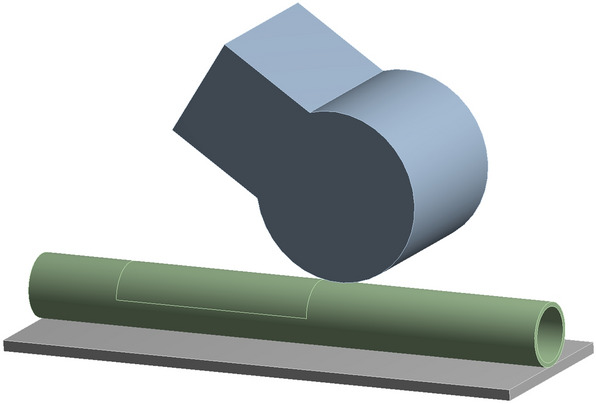
The model of a signal roller extruding from the hose.

**Figure 5:**
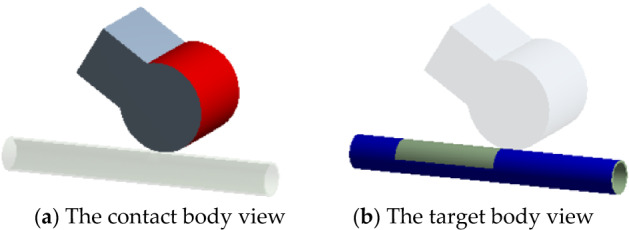
The contacts of the model.

To obtain the curve of *L* changing with the release time *t*, we established a model of a signal roller extruding the hose in Ansys19.0, as shown in Fig. 4. The model consisted of a base, a roller, a tube, and a flow. The geometry of the model is shown in Fig. 4. The contact body and the target body is shown in Fig. 5. The base and roller is structural steel. The tube is neoprene.

We set the initial conditions for the model analysis; the physical type is defined by the structure, and the analysis type is defined by transient. We selected 22 °C as the environment temperature. The step control is defined by time. The time step was chosen as a rough estimate based on Eq. (9).

9 $$ \Delta {\text{t = c}} \cdot \frac{\Delta x}{v} $$

where, *c* is the Courant number, $\Delta {\text{t }}$ is the grid size, *v* is the local velocity.

Bringing the grid size and velocity of this model into Eq. (9), the calculation is 0.0088 for a courant number of 1, so the step size is set to 0.001 s. The step end time was set to 0.3 s. We selected 65 RPM as the rotation speed and simulated the process of a roller squeezing a tube at the rated speed.

**Figure 6:**
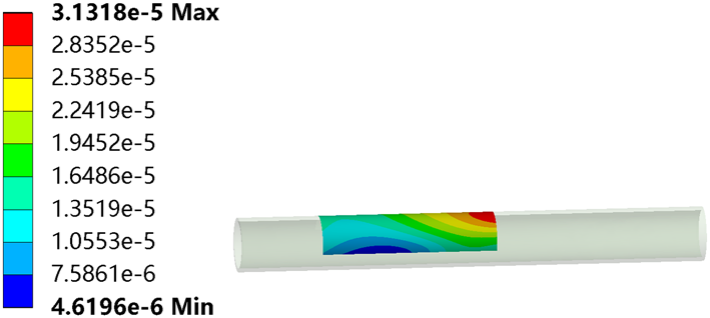
The selected area deformation of the tube.

After the simulation run, Fig. 6 shows the deformation of the selected area at the moment the hose starts to leave. It only shows the deformation of the entire hose selection area at this moment. The deformation of the same position at different moments will change.

**Figure 7:**
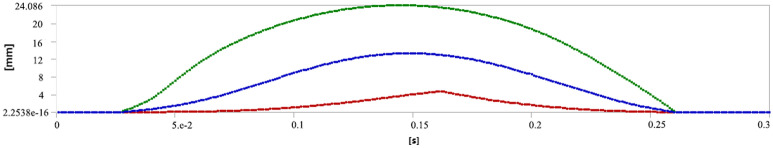
The total deformation curve of the selected area of the tube.

**Figure 8:**
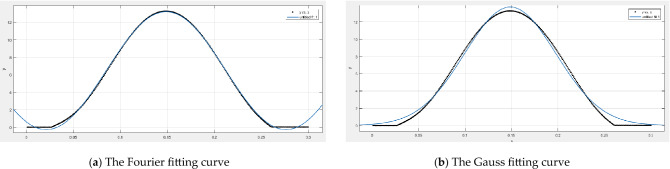
The fitting curve of the average deformation of a signal roller extruding the hose.

Figure 7 shows the curve of the rightmost position of the intercepted region with time. Red is the minimum deformation curve, green is the maximum deformation curve, and blue is the average deformation curve. It shows the deformation of the same position at different moments. We selected the average series data of total deformation and imported it into MATLAB for curve fitting, as shown in Fig. 8. We found that Gaussian and Fourier functions could fit the total deformation curve well. The results of the fitting functions are shown in Eqs. (10) and (11).

General model Fourier 1:

10 $$ f(x) = a0 + a1*\cos (x*w) + b1*\sin (x*w) $$

Coefficients (with 95% confidence bounds): a0 = 6.458 (6.432, 6.483); a1 = − 5.846 (− 5.894, − 5.799); b1 = − 3.348 (− 3.423, − 3.274); w = 24.63 (24.55, 24.71).

Goodness of fit: SSE:9.03; R-square: 0.9988; Adjusted R-square: 0.9988; RMSE: 0.1747.

General model Gauss 1:

11 $$ f(x) = a1*\exp ( - ((x - b1)/c1)^{2} ) $$

Coefficients (with 95% confidence bounds): a1 = 13.71 (13.6, 13.81); b1 = 0.1487 (0.1482, 0.1491); c1 = 0.06959 (0.06897, 0.07021).

Goodness of fit: SSE:49.74; R-square:0.9932; Adjusted R-square: 0.9932; RMSE: 0.4092.

Due to the high coefficient of determination of the Gaussian and Fourier curve fitting, both Gaussian and Fourier curves can be used as a function of the curve of the shell above the hose pump outlet. In practical applications, the shell processing forms such as casting or stamping can be selected for manufacturing and processing to achieve mass production.

In this study’s model, the radius of the rigid roller is 34.5 mm, the thickness of the hose is 15 mm, and the inner diameter is 25 mm. The distance from the center of the pump to the center of the roller is 94 mm. The geometric parameters of the model are brought into Eqs. (7) and (8),

then

12 $$ \alpha_{{{\text{max}}}} = {\text{arccos}}\left( {\frac{{R - {\text{d}}}}{R}} \right) = {\text{arccos}}\left( {\frac{{94 - 25}}{{{94}}}} \right) = 42.77^\circ $$

13 $$ {\text{t}}_{\max } = \frac{{\alpha_{{{\text{max}}}} }}{2\pi n} = \frac{42.77^\circ }{{2 \cdot 180^\circ \cdot \frac{65}{{60}}}}s \approx 0.11s $$

Due to the initial position delay, the Fourier function (9) in [0.027 s, 0.137 s] was chosen as a function of the curve of the shell above the hose pump outlet.

### Ethics approval

This article does not contain any studies with human participants or animals.

### Consent for publication

All authors agree to publication of the manuscript.

## Fluid–solid coupling simulation comparison test of the hose pump

To describe the model behavior of the hose pump accurately, the shell shape optimization of a hose pump requires an accurate model. There is a strong coupling relationship between pipe deformation and fluid flow, so fluid–structure interaction (FSI) analysis is needed^22–25^. However, due to the large deformation of the hose, the convergence of fluid and structure is difficult, and the amount of computation is extensive. The pump could be considered a multi-body model and used for dynamic simulations in engineering applications^26–28^. This paper mainly studies the three roller hose pump commonly used in the fertilization process. To verify the feasibility and effectiveness of the optimization method, we performed simulations to compare the conventional hose pump and the optimized hose pump.

### Model establishment

**Figure 9:**
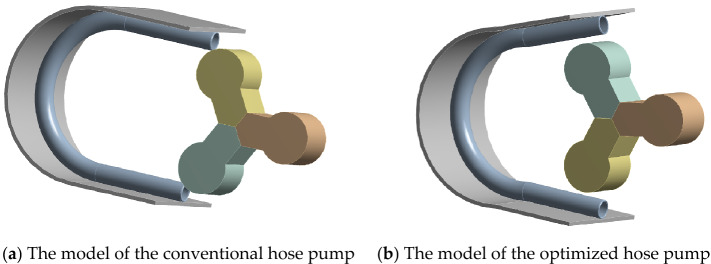
The hose pump models.

We established two three-dimensional, two-way fluid–structure coupling models of the hose pump, as shown in Fig. 9. Table 1 displays the model parameters of the hose pump. Then we established the computational domain for the numerical simulation.

**Table 1:** Geometry parameters of the hose pump.

Rotor speed	Number of rollers	Diameter of roller	Hose inlet diameter	Hose outlet diameter	The diameter of the bending part of the shell U-shaped plate	The width of the shell U-shaped plate	The thickness of the shell U-shaped plate	The flat length of the shell U-shaped plate
r/min	Pcs	mm	mm	mm	mm	mm	mm	mm
65	3	69	25	25	260	200	5	140

**Figure 10:**
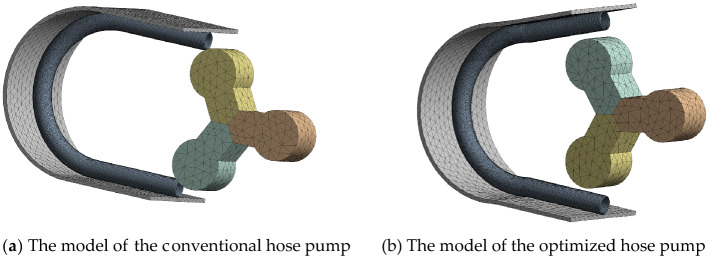
Grid generation.

To accelerate the calculation speed and maintain the iteration accuracy, we transported the geometry models of the hose pumps into ICEM 19.0 for meshing. As shown in Fig. 10, the unstructured tetrahedrons was generated by patch conforming for the roller and shell, the size of the grids is 0.015 mm. In contrast to the generation of the mesh of the roller and shell, the hexahedral structured grid is used in the hose, the size of the grids is 0.002 mm. The hose is refined with high grid numbers due to the sudden change in geometry. The total size of mesh finally used in calculation was 3.5 × 10^5^ after mesh independent check. It is vital to carry out a grid independence test on the generated meshes. We set up three monitoring points at the outlet and used the flow velocity as the reference quantity for grid independence. We conducted grid independence tests with grid numbers of 2.0 × 10^5^, 3.0 × 10^5^,4.0 × 10^5^ and 5.0 × 10^5^. It is found that when the grid number was greater than 3.0 × 10^5^, the difference in flow velocity at the monitoring points was small. Therefore, it is considered that the grid independence has been achieved when the grid number is 3.0 × 10^5^. Therefore the models grid meets the requirements of calculation accuracy and speed.

The conservation of mass and momentum are the governing equations in the FSI simulations. It is assumed that the flow squeezing through the roller is incompressible. The three-dimensional time-averaged Navier–Stokes equation (Reynolds equation) for incompressible turbulent flows. To simulate the actual flow of fluid in the rubber hose of the hose pump, we established turbulence modeling with realizable k-e. The realizable k-e model is defined as follows:

14 $$ \frac{{\partial \left( {\rho k} \right)}}{\partial t} + \frac{{\partial \left( {\rho ku_{i} } \right)}}{{\partial x_{i} }} = \frac{\partial }{{\partial x_{j} }}\left[ {\left( {\mu + \frac{{\mu_{t} }}{{\sigma_{k} }}} \right)\frac{\partial k}{{\partial x_{j} }}} \right] + G_{{\text{k}}} - \rho \varepsilon $$

15 $$ \frac{{\partial \left( {\rho \varepsilon } \right)}}{{\partial {\text{t}}}} + \frac{{\partial \left( {\rho \varepsilon u_{i} } \right)}}{{\partial x_{i} }} = \frac{\partial }{{\partial x_{j} }}\left[ {\left( {\mu + \frac{{\mu_{t} }}{{\sigma_{\varepsilon } }}} \right)\frac{\partial \varepsilon }{{\partial x_{j} }}} \right] + \rho C_{1} E\varepsilon - \rho C_{2} \frac{{\varepsilon^{2} }}{{k + \sqrt {v\varepsilon } }} $$

where *k* is the turbulent kinetic energy, $\varepsilon$ is the rate of dissipation, $\rho$ is the fluid density, $\mu_{{\text{t}}}$ is the fluid dynamic viscosity, and $\sigma_{{\text{i}}} ,\sigma_{\varepsilon } ,C_{2}$ is the closure factor.

16 $$ C_{1} = \max \left( {0.43,\;\frac{\eta }{\eta + 5}} \right) $$

17 $$ \eta = \left( {2E_{ij} \cdot E_{ij} } \right)^{1/2} \frac{k}{\varepsilon } $$

18 $$ E_{ij} = \frac{1}{2}\left( {\frac{{\partial u_{i} }}{{\partial x_{j} }} + \frac{{\partial u_{j} }}{{\partial x_{i} }}} \right) $$

19 $$ \mu_{1} = \rho C_{\mu } \frac{{k^{2} }}{\varepsilon } $$

20 $$ C_{\mu } = \frac{1}{{A_{0} + A_{s} U*k/\varepsilon }} $$

21 $$ \sigma_{i} = 1.0 $$

22 $$ \sigma_{\varepsilon } = 1.2 $$

23 $$ C_{2} = 1.9 $$

We describe the fluid in an arbitrary Lagrangian–Eulerian (ALE) framework. The ALE derivative induced by a deformation of the fluid domain, $\phi :{{{\Omega }_{0}}^{\text{f}}} \times \text{  { I}} \to R^{3}$ for a scalar function ${\text{f}}:{{{\Omega }_{0}}^{\text{f}}} \times \text{ { I}} \to R^{3}$ is defined as:

24 $$ \frac{{D^{\phi } f}}{Dt}: = \frac{\partial f}{{\partial t}} + \frac{\partial \phi }{{\partial t}} \cdot \nabla f $$

The deformed fluid domain is defined as $\forall t \in I$ as $\Omega _{t}^{\text{f}} :{ = \phi (\Omega }_{0}^{\text{f,t}} {)}$. We determined the pipe displacement via a neo-Hookean hyper-elastic model in a Lagrangian framework:

25 $$ \rho^{s} \frac{{d^{2} \eta^{2} }}{{dt^{2} }} + \rho^{s} \alpha \frac{{d\eta^{s} }}{dt} - \nabla {\text{ T}}^{s} - \beta \frac{d}{dt}\nabla {\text{ T}}^{s} = 0 $$

where $\uprho ^{s}$ is the displacement field, $\uprho ^{s}$ is the pipe density, $\text{T}^{s}$ is the stress tensor, $\upalpha$ is the mass damping coefficient, and $\upbeta$ is the stiffness damping coefficient.

Without considering the dissipation factor, the interaction between the pipe and the roller is frictionless. The near-wall treatment is standard wall functions. For the fluid part, the standard slip-free condition is adopted, and the wall roughness of all flow fields is smooth. The working fluid is water at 22 °C, and the density is $\uprho$ = 10^3^ kg/m^3^. The volume of the hose is almost constant when it is extruded, so it is classified as a hyper-elastic material^29,30^. The inlet boundary condition type is mass-flow-inlet. The turbulence specification method at the inlet is intensity and hydraulic diameter. The turbulence specification method at the outlet is intensity and viscosity ratio. The finite volume method is used to discretize the transport equations. The coupling between the pressure and velocity of the model pump is solved by the SIMPLE algorithm.

### Results and discussion

**Figure 11:**
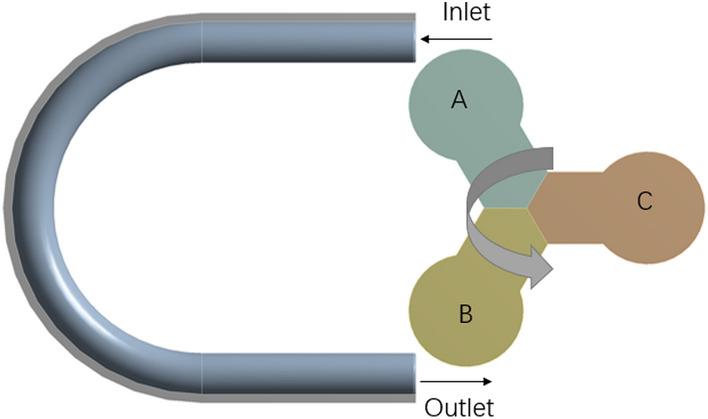
Initial position.

The speed of the hose pump was set to 65 r/min. The step control was defined by time, the time step was set to 0.002 s, and the end of step time was set to 2 min. Figure 11 shows the initial position. After 154 h, the simulation operation ended the run. During the actual assembly of the hose pump, the actual roller completely extruded the hose. The simulation must follow the principle of energy conservation to achieve good convergence, so there is a gap of 2.5 mm on the inner wall of the hose. According to the calculation, the flow velocity on the suction side of the fluid is about 0.6 m/s. In the simulation, the inlet velocity was set to 0.6 m/s, and the outlet pressure was set to 0 MPa. The roller rotates counterclockwise. The full three-dimensional flow process in the hose pipe of the hose pump was obtained via numerical simulation analysis.

#### Pressure distribution

**Figure 12:**
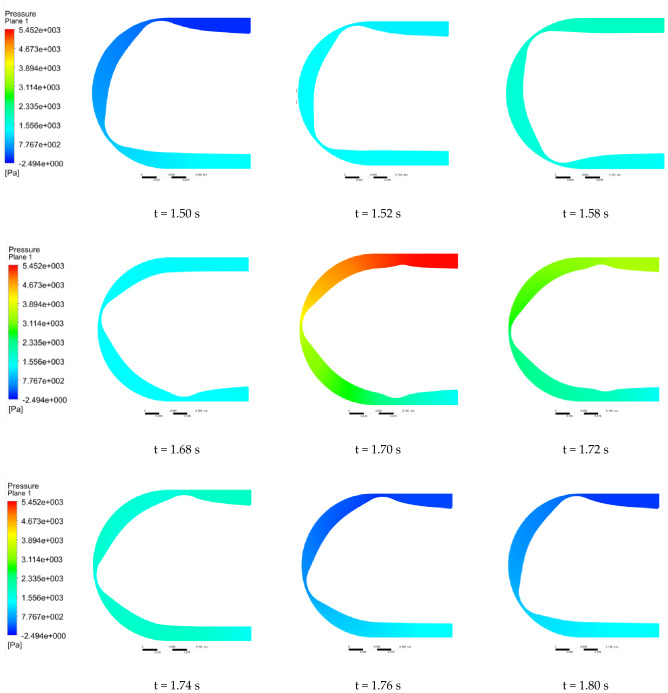
Pressure distribution of the conventional hose pump at different times.

**Figure 13:**
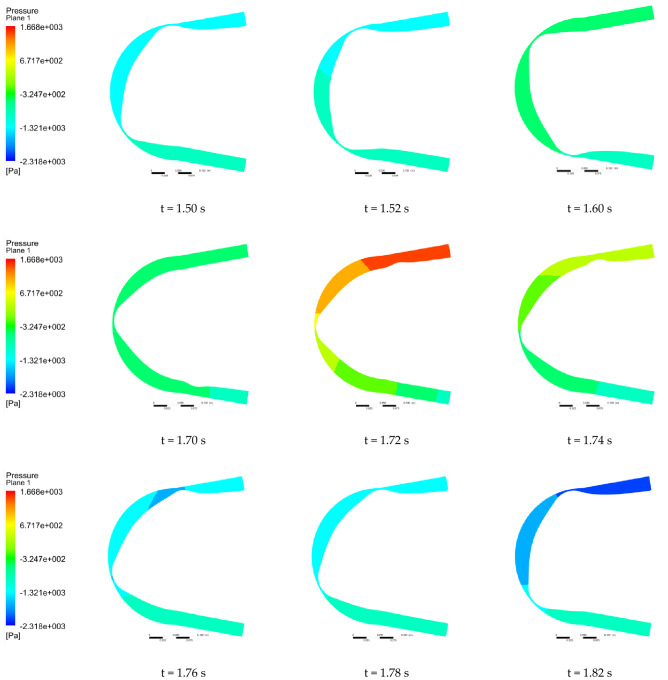
Pressure distribution of the optimized hose pump at different times.

We selected a cross section of the center of the hose to observe the pressure change. Figures 12 and 13 show the flow pressure distribution in one cycle for the conventional hose pump and the optimized hose pump. The simulation results show that the hose pump reached 65 HZ after 1.5 s, and the change period of flow field was 0.3 s. For the conventional hose pumps, at 1.50 s, roller A entered the hose pump and the fluid pressure in the inlet section and slowly increased. It demonstrated even slightly greater than the pressure in the outlet section at 1.58 s, then slowly decreased until 1.68 s. At 1.52–1.68 s, although the pressure changed, the overall range of change was low, so the fluid movement was in a relatively stable phase. The pressure in the inlet section reached a maximum at 1.70 s when roller B (in front of A) started to release the hose, and roller C (behind A) started to squeeze the hose. The pressure in the outlet section also increased due to the inlet pressure. Then the pressure in the inlet rapidly decreased to a minimum pressure at 1.76 s. Thus, the flow pressure in the tube changed the most during the process of the roller leaving and entering the hose pump, which was an unstable phase. The optimized pump pressure change process was similar, but with a 0.2 s delay, which may be due to the backward movement of the initial position of the roller squeezing the hose after the inlet shape optimization. The fluid pressure range of the traditional hose pump was (− 2.49, 5452.06) Pa, and the optimized hose pump fluid pressure range was (− 2318.23, 1668.21) Pa. This showed that the maximum fluid pressure decreased by 3783.85 Pa, and the minimum pressure increased by − 2315.74 Pa. The optimized hose pump flow pressure variation range was reduced by 26.92%, so the pressure in the whole operation process was more stable.

#### Velocity contour and streamline distribution

**Figure 14:**
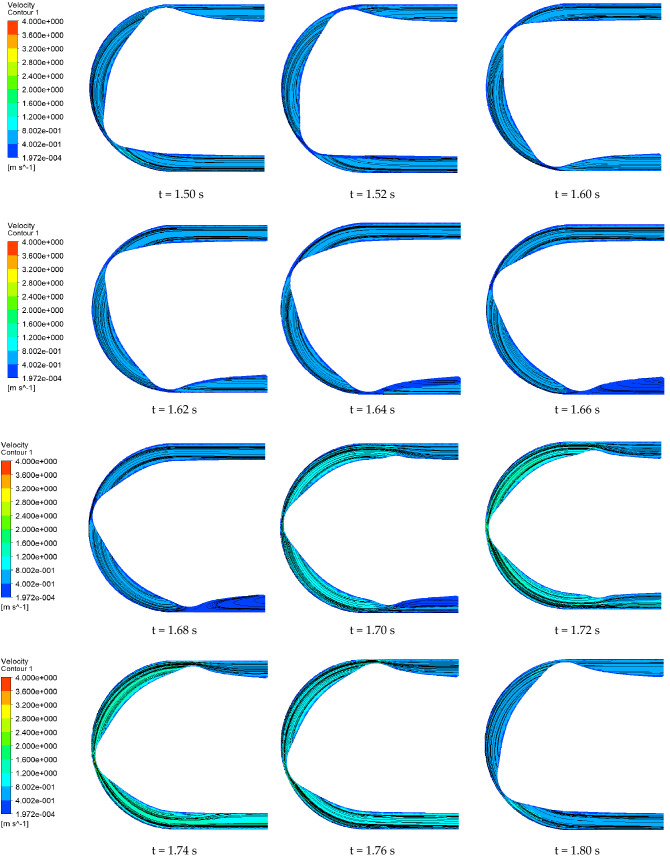
Velocity contour and streamline distribution of the conventional hose pump at different times.

**Figure 15:**
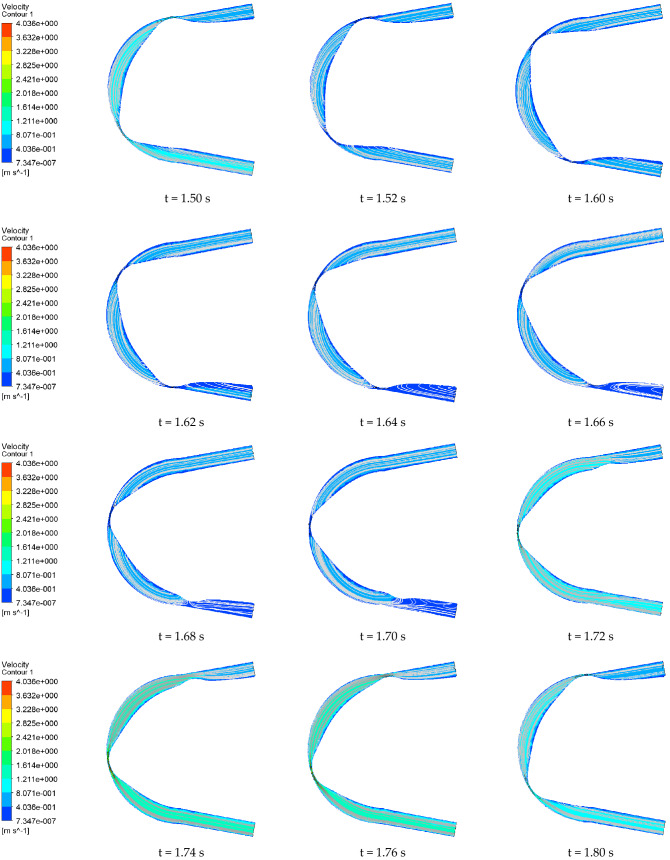
Velocity contour and streamline distribution of the optimized hose pump at different times.

Figures 14 and 15 show the velocity contours and streamline distribution of the conventional hose pump and the optimized hose pump in one cycle. For the conventional hose pump, the fluid velocity contours and streamlines were relatively stable at 1.50–1.62 s. When roller B (in front of A) started to release the hose, and roller C (behind A) started to squeeze the hose, the flow velocity contour and streamline at the outlet started to change greatly. The vortex shape of the flow velocity streamline was obvious at 1.64–1.70 s. This phenomenon increased the unstable flow in the pump. At 1.70 s, the flow velocity streamline at the outlet gradually recovered, whereas the flow velocity near the roller A increased. At 1.72–1.76 s, the flow velocity in the pump was higher than the other times. This is probably because the outlet and inlet are not blocked during this period. The optimized pump pressure change process was similar. The flow velocity range of the conventional hose pump was (0.00001972, 4.000) m/s, and the optimized hose pump flow velocity range was (0.00000007347, 4.036) m/s. The results thus showed that the flow velocity range of the optimized hose pump changed little. However, after comparison and calculation, we found that the average fluid flow velocity of the optimized hose pump increased by about 10% in one cycle.

#### Mass flow

The mass flow rate does not vary with time, space temperature, and pressure. To verify the effect on the flow output of the hose pump, we compared the inlet and outlet mass flow rates of the conventional hose pump and the optimized hose pump. The net mass flow rate of the conventional hose pump was 0.2877146 kg/s, whereas the net mass flow rate of the optimized hose pump was 0.3131438 kg/s. The mass flow rate of the optimized hose pump was 8.84% higher than that of the conventional hose pump, indicating that the optimized hose pump’s flow output performance was improved.

## Prototype test

**Figure 16:**
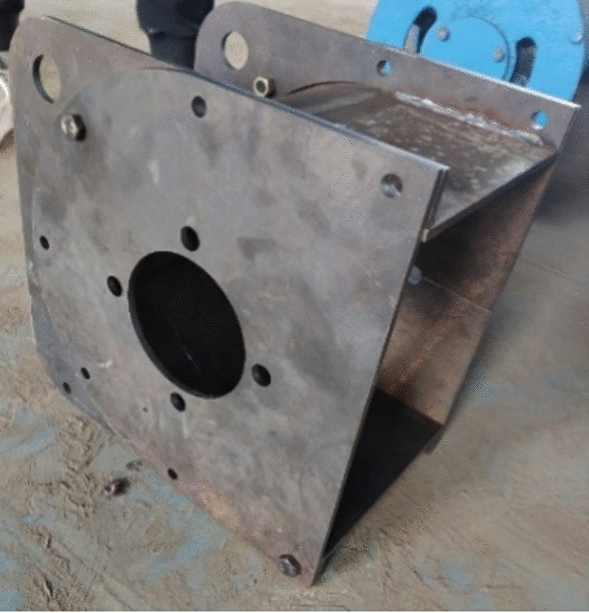
The optimized shell.

**Figure 17:**
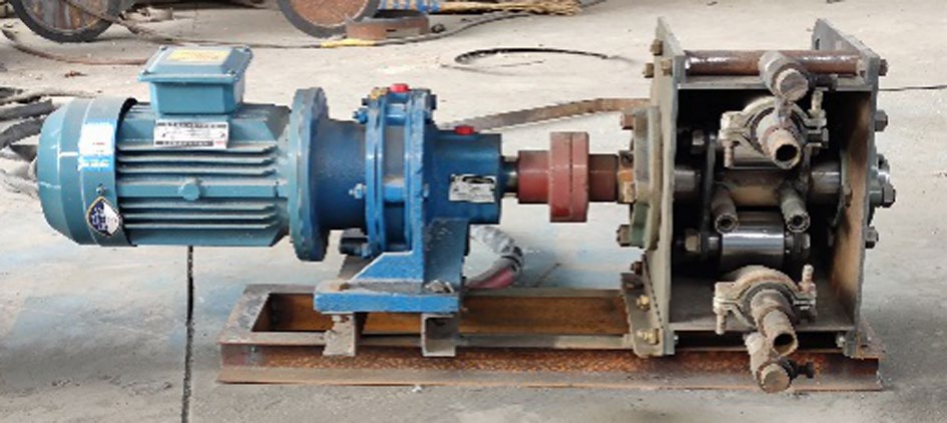
The optimized hose pump prototype.

To test the actual effect of the hose pump shell optimization, a prototype test was needed. We commissioned the shell, manufactured by a local company, as shown in Fig. 16, and then we assembled the hose pump prototype as a whole, as shown in Fig. 17.

**Figure 18:**
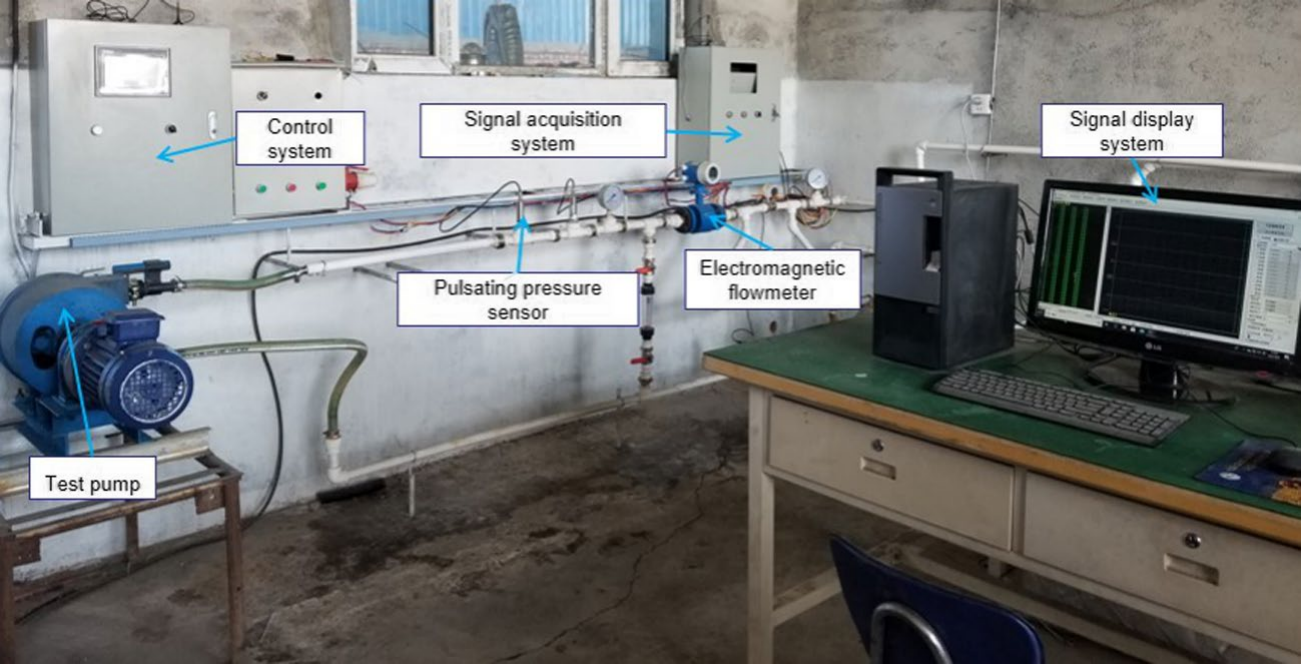
The test system of the hose pump.

We conducted a test in a test stand system, as shown in Fig. 18. We installed a high precision pulsation pressure sensor (measurement uncertainty ± 0.5%) in the outlet pipe to measure the pulsation pressure in the pipe and an electromagnetic flow meter (measurement uncertainty ± 0.5%) in the pipe to measure the flow change. We started the hose pump by following all standard start-up procedures and run it until proper operating conditions were reached. The data acquisition system collected data from the electromagnetic flow meter and the pulsating pressure sensor in real time and displayed them on the signal display system. We tested the conventional hose pump (shown in Fig. 18) and the optimized hose pump prototype separately and compared them. We considered repeatability during the experiments and conducted five replicate experiments, the measurement uncertainty according to Bessel’s formula is about 0.0025.

**Figure 19:**
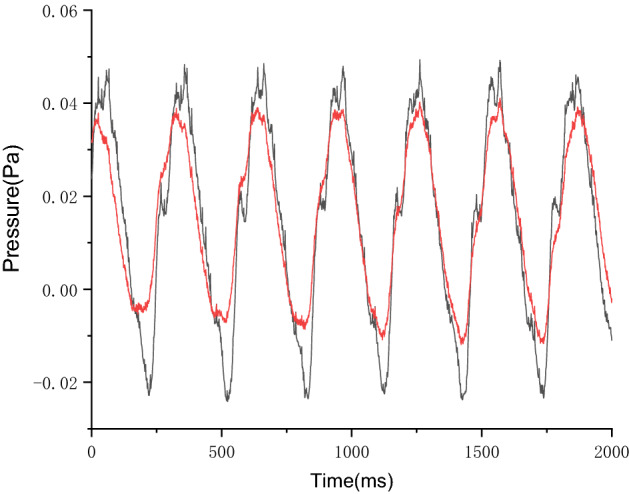
The pulsating pressure comparison in the outlet pipe at 65 Hz between the conventional hose pump and the optimized hose pump.

Because it was not possible to measure the pressure variation in the pump in real time, we observed the prototype test pulsating pressure variation in the outlet pipe. Figure 19 shows the variation in the pulsating pressure in the outlet pipe at operating frequencies of 65 Hz. Although the pulsating pressure varied, the pulsating pressure variation cycles of the conventional hose pump and the optimized hose pump at the same frequency remained unchanged. The pulsating pressure variation range of the optimized pump was reduced by about 20% from that of the conventional hose pump, which differed from the simulation results by 6.92%. The smaller range of pulsation pressure variation may be due to the smoother extrusion and release process of the hose pump after the shell optimization. Some deviations from the simulation results may be due to the external piping at the outlet being influenced by external pressures and other factors.

**Figure 20:**
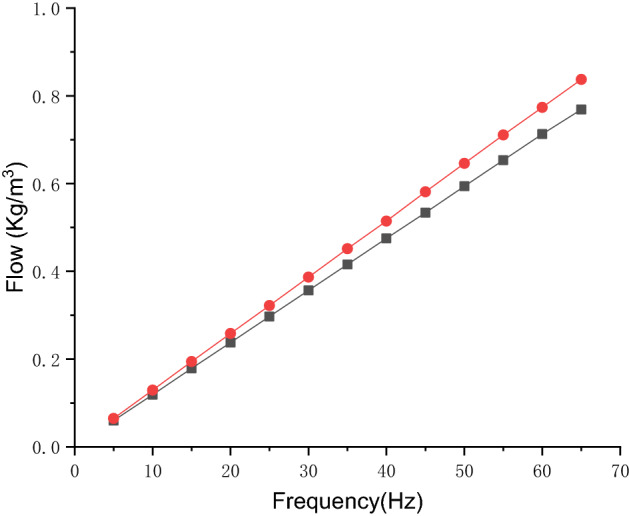
The flow comparison at different frequencies between the conventional hose pump (black) and optimized hose pump (red).

**Figure 21:**
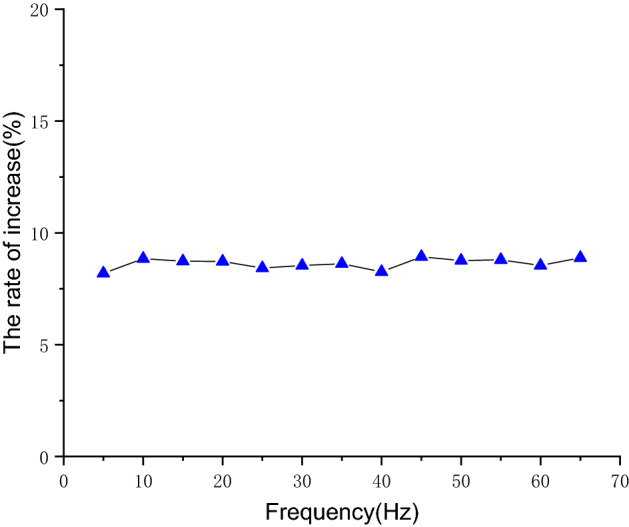
The rate of flow increase at different frequencies between the conventional hose pump and optimized hose pump.

Figure 20 shows the flow comparison of the hose pump at different operating frequencies. As can be seen, the optimized hose pump flow is higher than the conventional hose pump. The higher the operating frequency, the greater the difference in flow between the conventional hose pump and the optimized hose pump. In the actual operation of the hose pump, the speed of the hose pump is controlled by a frequency converter, and the frequency is proportional to the rotor speed. Equation (4) shows that the flow rate is proportional to the speed. The flow is proportional to the frequency, so the difference between the conventional hose pump and the optimized hose pump will increase with the increase of frequency. As shown in Fig. 21, experimental data show the flow of the optimized hose pump increased by about 8.63% at the different operating frequency. This may be due to the reduction of the flow reflux after shell optimization. Compared with the simulation results, the error between the test flow rate and the simulated flow output was within ± 3%.

## Conclusions

In this paper, we first analyzed the causes of pulsation and flow output characteristics of hose pumps then proposed a shell optimization method to reduce pulsation by using the squeeze curve as the outlet shell shape curve of the hose pump. We then conducted a simulation experiment to compare the flow variation of the optimized hose pump and traditional hose pump on the basis of fluid–solid coupling simulation. Finally, we conducted a prototype comparison test. The following are some main findings and suggestions, which can be considered in the process of hose pump design and manufacture to improve the operational reliability of hose pumps.

1. Gaussian function and Fourier function can well fit the curve of the roller releasing hose. The shell optimization method of using the extrusion curve as the shell shape curve of the outlet of the hose pump can be used to reduce pulsation.
2. The three-dimensional two-way fluid–structure coupling model of the hose pump can effectively simulate the fluid pressure and flow rate changes in the pump, which helps optimize the structure.
3. The shell optimization hose pump pulsation pressure variation range can be reduced by about 20% and flow increased by about 8%, indicating that the structure optimization method of this paper is effective, which can provide reference for hose pump manufacturers.

## Data Availability

The datasets used or analyzed during the current study are available from the corresponding author on reasonable request.

## Code availability

The numerical model software during the current study are available from the corresponding author on reasonable request.
